# Dual‐Vibration‐Assisted Charge Transport Through Hexabenzocoronene in Single‐Molecule Junctions

**DOI:** 10.1002/advs.202408310

**Published:** 2024-11-20

**Authors:** Miao Zhang, Boyu Wang, Hongxing Jia, Xinmiao Xie, Jie Hao, Li Zhou, Pingwu Du, Jinying Wang, Chuancheng Jia, Xuefeng Guo

**Affiliations:** ^1^ Center of Single‐Molecule Sciences Institute of Modern Optics Frontiers Science Center for New Organic Matter Tianjin Key Laboratory of Micro‐Scale Optical Information Science and Technology College of Electronic Information and Optical Engineering Nankai University 38 Tongyan Road, Jinnan District Tianjin 300350 P. R. China; ^2^ College of Materials Science and Engineering Chongqing University 174 Shazheng Street, Shapingba District Chongqing 400044 P. R. China; ^3^ Hefei National Research Center for Physical Sciences at the Microscale Anhui Laboratory of Advanced Photon Science and Technology CAS Key Laboratory of Materials for Energy Conversion Department of Materials Science and Engineering iChEM University of Science and Technology of China 96 Jinzhai Road Hefei Anhui 230026 P. R. China; ^4^ Beijing National Laboratory for Molecular Sciences National Biomedical Imaging Center College of Chemistry and Molecular Engineering Peking University 292 Chengfu Road, Haidian District Beijing 100871 P. R. China

**Keywords:** dual‐vibration‐assisted charge tunneling, graphene‐based single‐molecule junction, hexabenzocoronene

## Abstract

Gaining deep understanding and effective regulation of the charge transport mechanism within molecular junctions is essential for the development of electronic devices. In this work, a series of hexabenzocoronene‐based single‐molecule junctions are successfully constructed, and their temperature‐dependent charge transport properties are studied. It is found that rotational vibrations of both benzene and hexabenzocoronene rings are sequentially excited as the temperature increases, and the electron‐vibration coupling enhances charge tunneling. In addition, the transition temperature between distinct vibration‐assisted tunneling modes and the activation energies show strong correlations with the molecular vibration frequency and molecular length. This study unveils the distinct dual‐vibration‐assisted molecular tunneling mechanism, significantly enhancing the ability to precisely control molecular charge transport and develop functional molecular devices.

## Graphical abstract

Stable single‐molecule devices based on hexabenzocoronene oligomers are successfully fabricated through molecular engineering. In these junctions, torsional vibrations of both benzene and hexabenzocoronene rings are coupled with electrons, resulting in a dual‐vibration‐assisted charge tunneling transport.

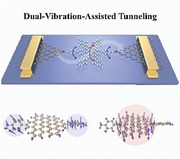

## 1 Introduction

As the traditional microelectronics technology approaches its physical limits, single‐molecule devices offer a promising new approach to surpass existing semiconductor‐based technologies, with the potential to achieve smaller device sizes and higher levels of integration.^[^ ^1^, ^2^ ^]^ At the single‐molecule level, charge transport no longer follows classical mechanisms, but involves non‐classical phenomena such as quantum tunneling^[^ ^3^, ^4^ ^]^ and quantum interference.^[^ ^5^ ^]^ By precisely controlling the charge transport behavior within single‐molecule junctions, it is possible to design novel molecular wires^[^ ^6^, ^7^ ^]^ and various molecular functional devices.^[^ ^8^, ^9^, ^10^ ^]^ Therefore, the exploration of charge transport mechanisms in single‐molecule junctions has become a core area of molecular electronics.

In most oligomeric molecular junctions, charge primarily transfers through a tunneling mechanism when the molecular chain is short, where molecular conductance decays exponentially with length.^[^ ^11^, ^12^ ^]^ Conversely, in longer molecular chains, the charge transfer mechanism is predominantly hopping,^[^ ^13^ ^]^ significantly influenced by temperature, as the thermal energy is able to excite the transported charges. In contrast, in the tunneling mechanism, electrons are generally considered to pass through the barrier through quantum tunneling, which is least affected by temperature.^[^ ^14^, ^15^, ^16^, ^17^ ^]^ Nevertheless, further studies have shown that temperature can modulate charge transport through mechanisms such as thermally assisted tunneling. In these mechanisms, higher temperatures can broaden the Fermi–Dirac distribution of the electrodes or excite the vibrational modes in the molecules, thereby modulating electron tunneling.^[^ ^18^, ^19^, ^20^, ^21^ ^]^ In the study of vibration‐assisted tunneling mechanisms, the relationship between molecular vibrations, molecular length, and charge transport remains unclear because molecules of the same length are usually used. To further explore the interactions among these factors, it is necessary to construct oligomeric molecular junctions with high rotational degrees of freedom.

Hexabenzocoronene (HBC), with its extensive π‐conjugated system, has become an efficient medium for charge transport in organic electronic devices.^[^ ^22^, ^23^, ^24^ ^]^ HBC molecules not only exhibit excellent thermal stability, but also can be chemically modified to adjust their electronic properties, showing unique advantages in the development of electronic devices with specific functions and long‐term stability.^[^ ^24^, ^25^, ^26^ ^]^ Previous research indicates that the primary mechanism of charge transport in HBC monomer is through quantum tunneling.^[^ ^27^ ^]^ In this work, we design a series of HBC oligomers with rotational degrees of freedom and fabricate single‐molecule junctions based on these oligomers using graphene‐molecule‐graphene single‐molecule junction (GMG‐SMJ) technique. By studying the temperature dependence of charge transport, the torsional vibrations of benzene and HBC rings and the influence of molecular length on the charge transport mechanism are deeply explored.

## 2 Results and Discussion

**Figure 1:**
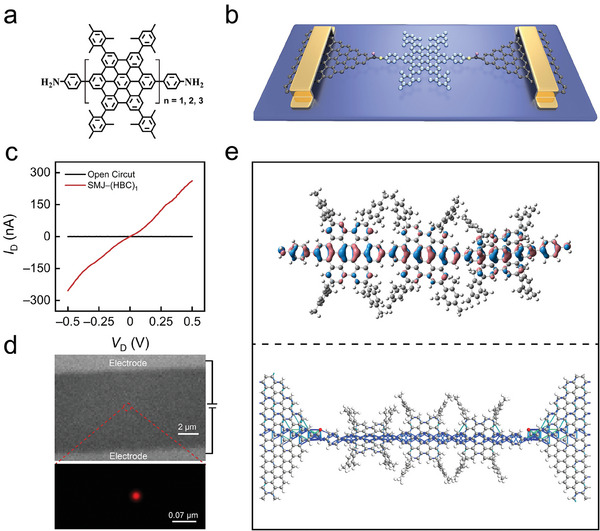
Structure characterization and electrical characterization of graphene−(HBC)*_n_*−graphene single–molecule junctions. a) Schematic diagram of chemical formula structure of HBC oligomers. The amino groups at the molecular ends serve as anchoring groups. b) Schematic diagram of (HBC)*_n_*‐based single‐molecule device, where HBC oligomers are covalently bonded to graphene electrodes via amide linkages. c) Current‐voltage (*I*_D_−*V*_D_) characteristic curves of the molecule before (black line) and after (red line) connection with the electrodes. *V*_D_, source‐drain bias; *I*_D_, source‐drain current. d) Super‐resolution fluorescence imaging of single molecule junction (graphene−(HBC)_1_−graphene) is obtained by stochastic optical reconstruction microscopy (STORM). e) Theoretical calculations of the electronic properties of graphene–(HBC)*_n_*–graphene junctions. HOMO orbital charge distribution of the (HBC)_3_ molecule (Top). The charge transmission path of the *p–*HOMO for the graphene*–*(HBC)_3_ *–*graphene junctions (Bottom).

The structures of the HBC oligomers are sketched in **Figure** **1**a, denoted as (HBC)*_n,_* where *n* represents the number of HBC units (1, 2, and 3, respectively). The ends of the HBC are linked to the benzene rings through sigma (σ) bonds. The introduction of σ‐bonds increases the rotational freedom between the phenyl and HBC rings. These molecules are successfully synthesized and introduced into graphene nanogaps to construct single‐molecule junctions (Figure 1b). Details of molecular synthesis and device fabrication are provided in Supporting Information (Schemes S1–S2 and Figures S1–S10, Supporting Information). These graphene‐(HBC)*_n_*‐graphene junctions exhibit good stability, attributed to covalent contacts via amide linkages. The response recovery of the current‐voltage (*I*_D_−*V*_D_) curves indicates a successful connection between the molecule and the electrode (Figure 1c; Figure S11, Supporting Information). Meanwhile, the distinct C═O (≈220 mV) and N─H (≈450 mV) stretching vibrations observed in the inelastic tunneling spectra (IETS) provide strong evidence for the covalent connection between HBC and graphene electrode (Figure S12, Supporting Information).^[^ ^28^, ^29^, ^30^ ^]^ In addition, the successful connection of a single molecule is further demonstrated using a super‐resolution fluorescence microscope.^[^ ^31^ ^]^ As illustrated in Figure 1d and Figure S13 (Supporting Information), the presence of only one bright spot between the electrodes confirms that only one molecule is connected across this pair of electrodes.

Recent research indicates that thermally induced rotational vibrations of benzene rings cause the charge transport mechanism in molecules to shift from temperature‐independent coherent tunneling at low temperatures to temperature‐dependent incoherent tunneling at higher temperatures.^[^ ^19^, ^21^ ^]^ These vibrations are thermally excited around 90 K, setting the transition temperature for this behavior around 90 K, although this is also influenced by the overall molecular structure. In the HBC molecular structure, HBC rings are directly connected to benzene rings via σ–bonds. Therefore, it is anticipated that at specific temperatures, the rotational vibrations of both benzene and HBC rings are thermally excited, necessitating consideration of the impact of these two rotational modes on charge transport. The trimethylphenyl groups attached to the periphery of each HBC unit enhance solubility in the solution, thus facilitating molecular assembly with electrodes. Since these groups are not located on the main transport channel of the *p*–HOMO (Figure 1e), their vibrations have a relatively minor impact on charge transport.

**Figure 2:**
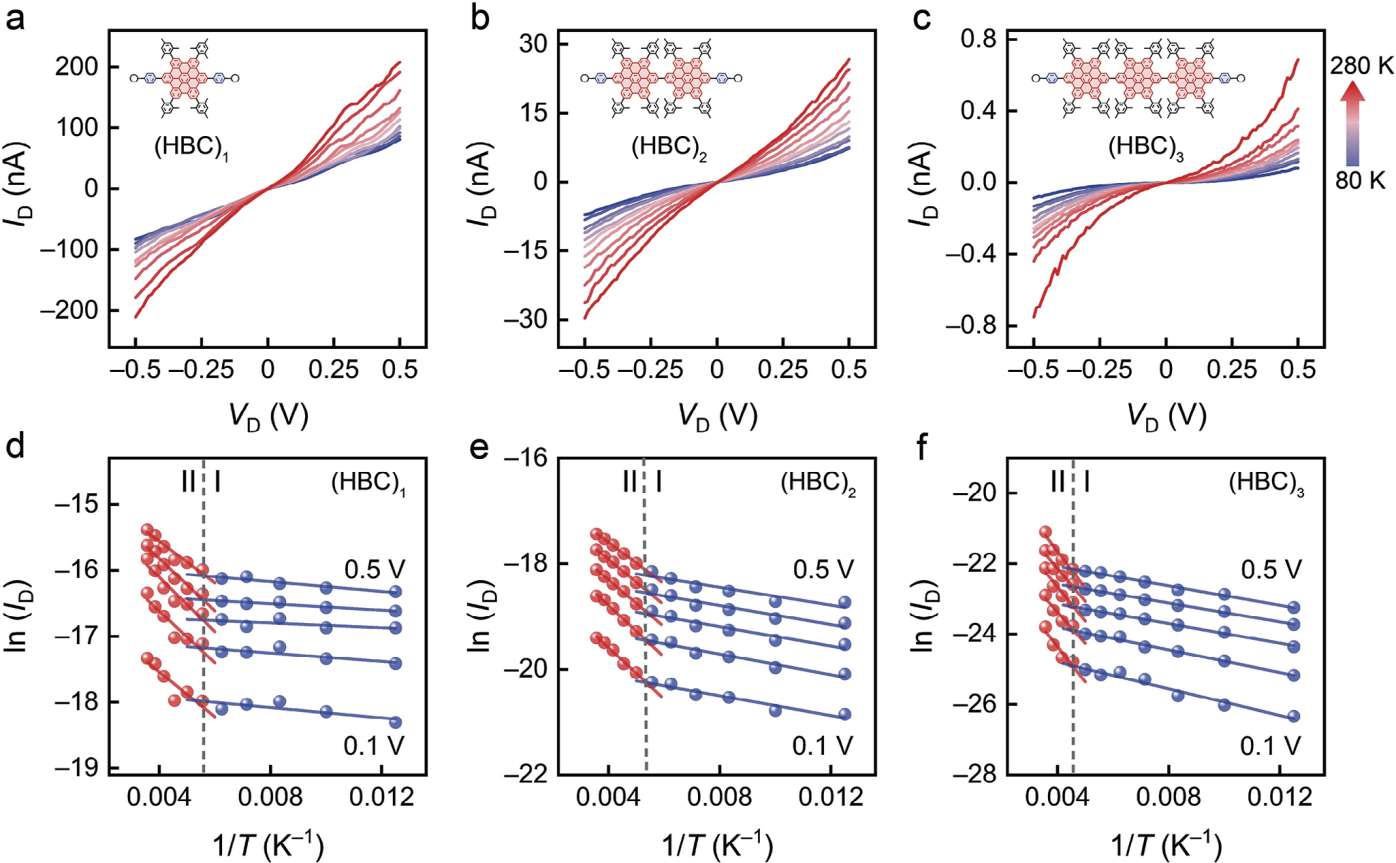
Temperature–dependent charge transport characteristics. a−c) *I*_D_−*V*_D_ curves of graphene–(HBC)*_n_*–graphene (*n* = 1, 2, 3, respectively) single–molecule junctions in the temperature range of 80–280 K. d–f) Arrhenius plots of ln (*I*_D_) versus 1/*T* for HBC junctions when *V*_D_ increases from 0.1 to 0.5 V with steps of 0.1 V.

The current through the molecular junction is measured across a temperature range from 80 to 280 K to investigate the effect of molecular vibrations on the charge transport behavior of (HBC)*_n_* junctions. The bias voltage is maintained between −0.5 and 0.5 V to ensure the stability of the single‐molecule junction. Within this range, the direct influence of the bias on charge transport is negligible, providing reliable and accurate experimental investigations of temperature effects. **Figure** **2**a−c displays the temperature–dependent current‐voltage (*I*_D_−*V*_D_) characteristic curves of the graphene‐(HBC)*_n_*‐graphene (*n* = 1, 2, 3, respectively) junctions. Their current increases with rising temperature, indicating characteristics of incoherent charge transport through the molecular junction. In a scanning tunneling microscope‐based break junction (STM‐BJ) experiment involving HBC, researchers observe that molecular conductance is insensitive to temperature changes within the range from 268.15 to 313.15 K,^[^ ^27^ ^]^ which appears to contradict our results. This discrepancy may be attributed to the dynamic nature of the STM‐BJ technique, whose resolution may not be sufficient to detect subtle structural variations between different units within the molecule. The effects of molecular vibrations are minimal and can be obscured by the broad half‐width of the conductance peaks, resulting in a weak dependence of conductance on temperature. The strong conjugation of the HBC molecule, along with the favorable alignment of the HOMO energy level with the electrodes and the exponential attenuation of molecular junction conductance with molecular length (Figure S14, Supporting Information), indicates that tunneling is the predominant charge transport mechanism in these molecular junctions. Therefore, it can be speculated that this incoherence stems from thermally activated molecular vibrations, where the molecular vibrations interact with charges to facilitate charge tunneling.

**Figure 3:**
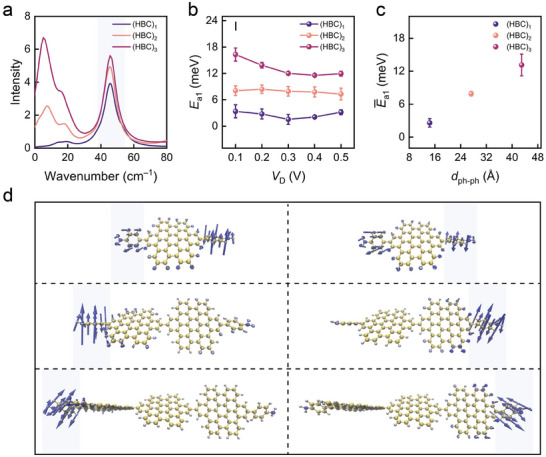
Phenyl vibration–assisted charge transport in graphene−(HBC)*_n_*−graphene junctions (*n* = 1, 2, 3). a) Theoretical calculation of vibrational spectra for HBC oligomers. The peaks in the blue‐shaded areas correspond to the vibrations of the benzene ring. b) Activation energy of charge transport in temperature region I (*E*_a1_) for (HBC)*_n_* (*n* = 1, 2, and 3) single–molecule junctions under different bias voltages *V*_D_. c) Variation of average activation energy *E̅*_a1_ with respect to *d*_ph–ph_ for graphene−(HBC)*_n_*−graphene (*n* = 1, 2, 3) junctions when 0.1 V ≤ *V*_D_ ≤ 0.5 V. d) Vibrational modes of phenyl twisting in (HBC)*_n_* (*n* = 1, 2, 3).

It is noteworthy that on the *I*_D_−*V*_D_ curve, the current increases more slowly with temperature in the lower temperature range, while it increases more rapidly in the higher temperature range (Figure 2a−c). Consequently, we perform piecewise fitting of ln (*I*_D_) versus 1/*T* (Figure 2d−f). There are primarily two temperature regions, delineated by ≈200 K. The slope of ln (*I*_D_)–1/*T* relationship is smaller in the low‐temperature region I, while it is larger in the high‐temperature region II. Repeated *I*_D_−*V*_D_ tests exhibit similar results (Figure S15, Supporting Information). This indicates that the activation energy for charge transport varies between these two regions, which may be related to the vibrations of different molecular units. To determine whether these two thermally activated charge transport mechanisms originate from molecular vibrations, we simulate the vibrational spectra of the molecule using first‐principles calculations. As illustrated in **Figure** **3**a, a prominent vibrational peak at ≈45–46 cm^−1^ corresponds to the torsional vibrational modes of the benzene rings on both sides of the molecule (Figure 3d). The energy of this vibration is close to the thermal energy at ≈66 K. This suggests that when the temperature exceeds ≈66 K, the vibrations of the benzene rings in the molecule can be activated, coupling with electrons to facilitate charge tunneling, a process known as vibration‐assisted tunneling.^[^ ^19^, ^21^ ^]^

Furthermore, the activation energy (*E*_a1_) for charge transport in (HBC)*_n_* molecular junctions within the low–temperature region I is calculated according to the Arrhenius equation $E_{a}=-\left(\frac{1}{k_{B}}\right)\left[\frac{d(\ln I)}{d(\frac{1}{T})}\right]$, where *k*_B_ is the Boltzmann constant. Figure 3b displays the characteristic curves of *E*_a1_ versus *V*_D_ for these (HBC)*_n_* molecular junctions. The *E*_a1_ of (HBC)_1_, (HBC)_2_, and (HBC)_3_ molecular junctions show little dependence on the bias voltage. The average activation energies (*E̅*_a1_) over the bias range of 0.1 to 0.5 V are calculated as 2.59 ± 0.76, 7.90 ± 0.40, and 13.14 ± 1.98 meV, respectively (Figure 3c). The *E̅*_a1_ increases with the number of HBC units, despite the phenyl‐torsion frequencies of (HBC)*_n_* remaining nearly constant. In incoherent tunneling, charge transport primarily depends on two factors: the static tunneling width (approximately the molecular length *L*), which leads to an exponential attenuation of current ($I=I_{0}^{\prime }\;e^{-\beta L}$); and the activation energy ($I=I_{0}\;e^{-E_{a}/\left(k_{B}T\right)}$), which is influenced by the energy barrier between the molecular orbital and the electrodes’ Fermi level, the orbital's coupling with electrodes, and electron‐vibration interactions. These relations cause the activation energy to be a function of molecular length. As illustrated in Figure 3d, in (HBC)_1_, the shorter distance between the benzene rings on both sides enhances the vibrational coupling, leading to gradual vibrational degeneracy under similar conditions. In (HBC)_2_ and (HBC)_3_, the increased distance between the central benzene rings causes the vibrational modes of the benzene rings on both sides to become more independent, gradually weakening the vibrational coupling. As the molecular length increases, the orbital‐electrode coupling weakens, tunneling probability decreases, and vibrations become more localized, resulting in a higher effective tunneling barrier or greater activation energy for charge transport.

**Figure 4:**
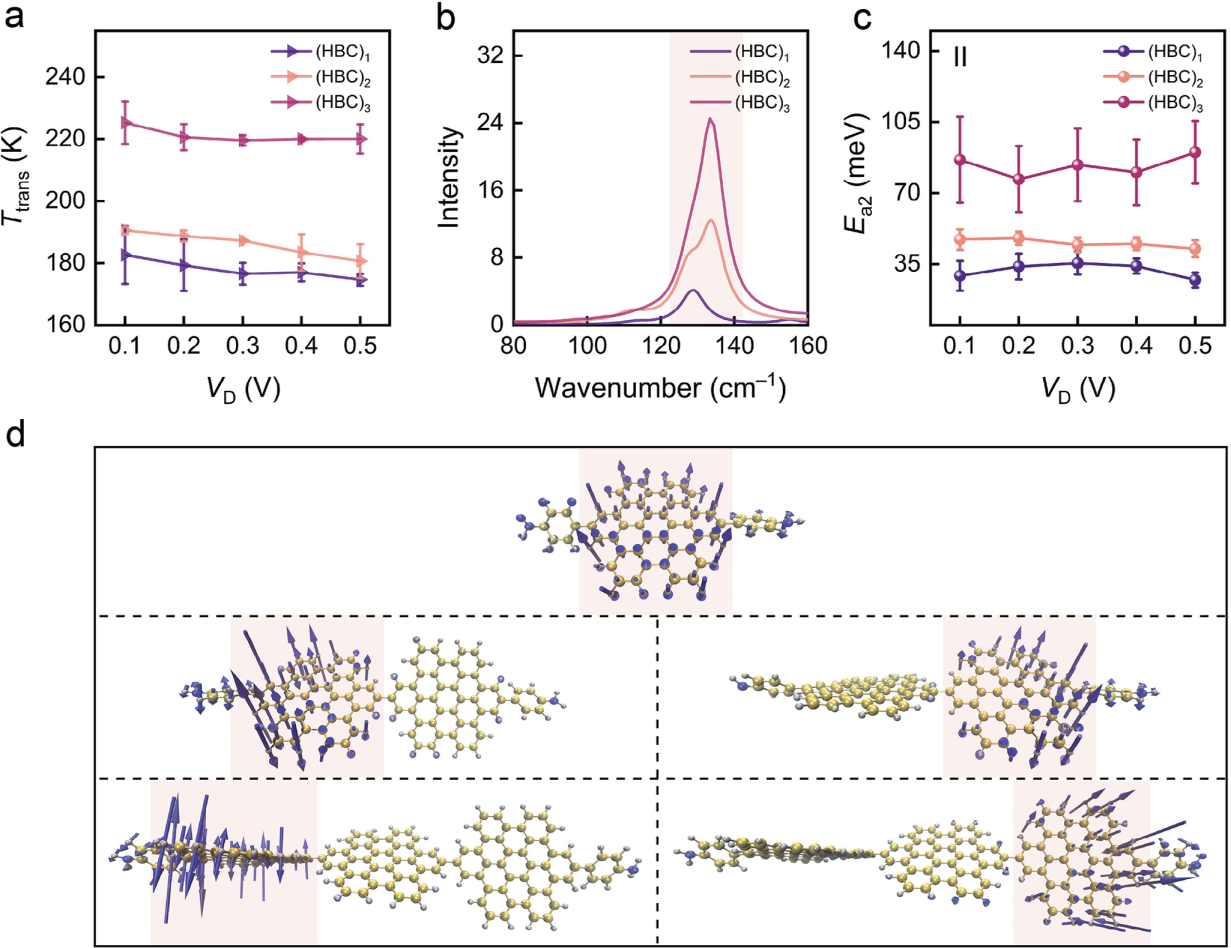
HBC ring vibration–assisted charge transport. a) *T*_trans_ (with error bars) for charge transport transitions from region I to region II within *V*_D_ = 0.1–0.5 V. b) Theoretical calculation of vibrational spectra for HBC oligomers. The peaks in the red‐shaded areas correspond to the vibrations of the HBC ring. c) Charge transport activation energy in temperature region II (*E*_a2_) for (HBC)_1_, (HBC)_2_, and (HBC)_3_ single–molecule junctions. d) Vibrational modes of one HBC ring in (HBC)_1_, (HBC)_2_, and (HBC)_3_.

When the temperature rises to the vicinity of 200 K, a significant transition in the charge transport characteristics of (HBC)*_n_* molecular junctions can be clearly observed (Figure 2d–f). To further investigate the transport mechanism in region II, we first calculate the transition temperature (*T*_trans_) at which the charge transport characteristics shift from region I to region II (**Figure** **4**a). The calculation involves extending the fitted lines of region I and region II as shown in Figure 2d–f, and identifying the intersection point as *T*_trans_. It is also observed that the transition temperature of HBC molecular junctions shows little dependence on the bias voltage. The *T*_trans_ values for (HBC)_1_, (HBC)_2_, and (HBC)_3_ molecular junctions are ≈180, 190, and 220 K, respectively. The thermal energy at 180–190 K corresponds closely to the torsional vibration energies of the HBC rings at frequencies of 128–134 cm^−1^ in the vibrational spectra (Figure 4b,d). Compared to the previous phenyl torsion peaks, the subsequent vibrational peaks in the molecular vibrational spectra are attributed to HBC torsional vibrations. This correlation suggests that the transition temperatures for charge transport characteristics observed in (HBC)_1_ and (HBC)_2_ align well with the phenyl and HBC torsions. At the transition temperature, the torsional vibrations of the HBC rings in the molecular backbone are excited, causing the charge transport to shift from benzene‐assisted tunneling to HBC‐assisted tunneling. Theoretical calculations indicate that the vibrational frequency of the HBC rings in (HBC)_2_ and (HBC)_3_ remains constant at ≈134 cm^−1^. However, in actual experiments, the transition temperature of (HBC)_3_ is 30 K higher than that of (HBC)_2_, which may be related to the simplified simulations of vibrational spectra of (HBC)_n_ by omitting trimethylbenzene groups (see more details below). Furthermore, the calculated vibrational spectra of the HBC connected to graphene fragments demonstrate that graphene electrodes have a minimal influence on the vibrations of benzene and HBC rings (Table S1, Supporting Information).

In the initial theoretical calculation model, we excluded the side chain (trimethylbenzene) because it is not located in the main conductive channel and has minimal impact on charge transport. Additionally, the presence of trimethylbenzene complicates the separation of the vibrations between the benzene and HBC rings. However, comparative calculations of the vibrational spectra of the HBC molecule, including all side chains, reveal a splitting of the HBC torsional peak in (HBC)_3_ and the appearance of a strong vibrational peak at ≈144 cm^−1^. Consequently, this results in a sharp increase in the transition temperature. Meanwhile, the *E*_a2_ of (HBC)_2_ and (HBC)_1_ remains similar, with the main vibrational peak at 128 to 134 cm^−1^ (Figure S16, Supporting Information). This confirms that the charge transport in the high‐temperature region II is assisted by HBC torsion. We further calculate the activation energy (*E*_a2_) for charge transport in high‐temperature region II and plot the characteristic curve of *E*_a2_ as a function of bias voltage (Figure 4c). The results show that in the process of vibration‐assisted charge transport, the activation energy gradually increases with molecular length. This indicates that the vibration‐assisted molecular tunneling is influenced by both molecular length and vibrations.

## 3 Conclusion

In summary, the charge transport mechanism of HBC oligomers is studied by constructing graphene−(HBC)*_n_*−graphene (*n* = 1, 2, 3) single‐molecule junctions. The torsional vibrations of benzene and HBC rings within the molecular backbone are sequentially thermally excited as the temperature increases from liquid nitrogen temperature to room temperature. These vibrations couple with electrons, resulting in dual‐vibration‐assisted incoherent tunneling, characterized by a nonlinear temperature dependence of currents in all HBC junctions. The transition temperature of (HBC)*_n_* junctions around 200 K corresponds to the vibrational energies of (HBC)*_n_* molecules, and the activation energies for charge transport depend on both molecular length and vibrations. Our findings deepen the understanding of molecular charge transport mechanisms, which is crucial for the development of molecular electronics.

## 4 Experimental Section

### Molecular Synthesis

See Section S1 (Supporting Information) for detailed information.

### Device Fabrication

A 25 µm thick copper foil was immersed in acetic acid for 15 min to remove surface oxides. After drying, the copper foil was placed in a tube furnace to grow high‐quality monolayer graphene on its surface via chemical vapor deposition. The graphene film was then transferred from the copper foil to a silicon substrate with a 300 nm thick SiO_2_ layer using the wet transfer method. Photolithography and deposition techniques were employed to create marks on the graphene surface. Subsequently, the graphene was processed into ribbons with 40 × 200 µm in the center of the device using photolithography and oxygen plasma etching techniques. Finally, 8 nm chromium (Cr) and 60 nm gold (Au) were thermally evaporated onto the graphene ribbons to fabricate metal electrodes, resulting in a graphene FET array device (Figures S7 and S8a, Supporting Information). Carboxylic acid‐terminated graphene nanoelectrodes were prepared on the ribbons using a dash‐line lithography method^[^ ^32^ ^]^ (Figures S8b and S10, Supporting Information). The carboxyl groups at the ends of electrodes form amide covalent bonds with amino groups at the ends of HBC molecules in an anhydrous and oxygen‐free environment, using pyridine as the solvent. The molecular solution concentration was maintained at 10^−4^ m, and the reaction was facilitated by the dehydrating agent, 1‐ethyl‐3‐(3‐dimethylaminopropyl) carbodiimide (EDCI, 25 mg), with a reaction time of 48 h (Figure S10, Supporting Information).

### Device Measurements

Before molecular bridging, conductance measurements were taken for each electrode pair within the device array using a Karl Suss PM5 probe station and a Keysight B1500A semiconductor device analyzer, with recorded levels ranging from 10^−3^ to 10^1^ pA. Following the molecular bridging, the test was repeated. A significant increase in conductance to the nA range indicated a successful molecular connection between the graphene electrodes. The Karl Suss PM5 station and the Keysight B1500A were employed for *I*–*V* measurements at room temperature, while for variable temperature conditions, the Lakeshore TTPX station and the Keysight B1500A were used within a vacuum environment. The bias voltage for *I*−*V* measurements remained consistent at a 0.01 V scan interval.

### Theoretical Calculations

The structures of all HBC oligomers were optimized using density functional theory (DFT) with a hybrid functional and the 6–31G(d) basis set. Empirical dispersion corrections were applied at the GD3(BJ) level, as implemented in the Gaussian 16 software package. Vibrational frequency calculations were performed under the same conditions. To assess the vibrational modes of HBC oligomers’ condensed rings without the influence of lateral groups, which have a limited impact on the transport pathway, the molecular structures were simplified by removing the lateral trimethylphenyl rings. The transmission pathway of the molecular junction was calculated using DFT within the nonequilibrium Green's function (NEGF) formalism, employing full settings.

## Conflict of Interest

Authors declare no competing interests.

## Author Contributions

X.G., C.J., J.W. and P.D. conceived and designed the experiments. M.Z. fabricated the devices and performed the device measurements. H.J. did the molecular synthesis. B.W. performed the theoretical calculations. X.G., C.J., J.W., P.D., M.Z., B.W., H.J., X.X., J.H. and L.Z. analyzed the data and wrote the paper. All the authors discussed the results and commented on the manuscript.

## Supporting information



Supporting Information

## Data Availability

The data that support the findings of this study are available from the corresponding author upon reasonable request.
