# Supplementary material for: Dual‐Vibration‐Assisted Charge Transport Through Hexabenzocoronene in Single‐Molecule Junctions
Source: Adv Sci (Weinh). 2024 Nov 20;12(2):2408310. doi: 10.1002/advs.202408310 (PMC11727374; doi:10.1002/advs.202408310)
Supplement: Supplementary file 1 — Supporting Information [file ADVS-12-2408310-s001.pdf]

ADVANCED SCIENCE
Open Access

Supporting Information

for *Adv. Sci.*, DOI 10.1002/advs.202408310

Dual-Vibration-Assisted Charge Transport Through Hexabenzocoronene in Single-Molecule Junctions

Miao Zhang, Boyu Wang, Hongxing Jia, Xinmiao Xie, Jie Hao, Li Zhou, Pingwu Du, Jinying Wang*, Chuancheng Jia* and Xuefeng Guo**

Supplementary Information for

Dual-vibration-assisted charge transport through hexabenzocoronene in single-molecule junctions

Miao Zhang[1,2], Boyu Wang[1], Hongxing Jia[3,4], Xinmiao Xie[2], Jie Hao[1], Li Zhou[1], Pingwu Du[3]*, Jinying Wang[1]*, Chuancheng Jia[1,2]* & Xuefeng Guo[1,2]*

[1]Center of Single-Molecule Sciences, Institute of Modern Optics, Frontiers Science Center for New Organic Matter, College of Electronic Information and Optical Engineering, Nankai University, 38 Tongyan Road, Jinnan District, Tianjin 300350, P. R. China.

[2]Beijing National Laboratory for Molecular Sciences, National Biomedical Imaging Center, College of Chemistry and Molecular Engineering, Peking University, 292 Chengfu Road, Haidian District, Beijing 100871, P. R. China.

[3]Hefei National Research Center for Physical Sciences at the Microscale, Anhui Laboratory of Advanced Photon Science and Technology, CAS Key Laboratory of Materials for Energy Conversion, Department of Materials Science and Engineering, iChEM, University of Science and Technology of China, 96 Jinzhai Road, Hefei, Anhui Province 230026, China.

[4]China College of Materials Science and Engineering, Chongqing University, 174 Shazheng Street, Shapingba District, Chongqing 400044, China.

*Corresponding authors. E–mail: guoxf@pku.edu.cn (X.G.); jiacc@nankai.edu.cn (C.J.); wangjynk@nankai.edu.cn (J.W.); dupingwu@ustc.edu.cn (P.D.)

Table of Contents

1. Molecular synthesis
2. Device fabrication
3. Device measurements
4. Characterization of single-molecule junctions
5. Charge transport characteristics

1. Molecular synthesis

$(HBC)_1$ compounds are synthesized based on the literature published.[1] The synthesis route of $(HBC)_2$ and $(HBC)_3$ compounds are shown in Scheme S1.

Due to the amino groups at both ends of the molecule are prone to oxidation, they are protected with *t*-butyloxycarbonyl (BOC) groups during synthesis. Before the molecule is connected to the electrodes, a further reaction is performed to remove the BOC groups. Take $(HBC)_1$ for example，The specific reaction route is shown in Scheme S2.

Materials: All chemicals are purchased from Innochem or Sigma-Aldrich and used without further purification. All organic solvents are purchased from Innochem or China Medicine Shanghai Chemical Reagent Co.

Representation: ^{1}H NMR spectroscopy: The reagent is $CDCl_3$, the instrument is the XR-400 nuclear magnetic resonance spectrometer from Bruker, the frequency of the applied magnetic field is 400 MHz, and the resolution is less than or equal to 0.2 Hz. High-resolution mass spectrometry: The instrument used is a Fourier transform high-resolution mass spectrometer from Bruker (model: SolariX XR), the ion source is an electron spray ionization (ESI) source, the measurement error is less than 1 ppm, and the resolution could reach 1000000.

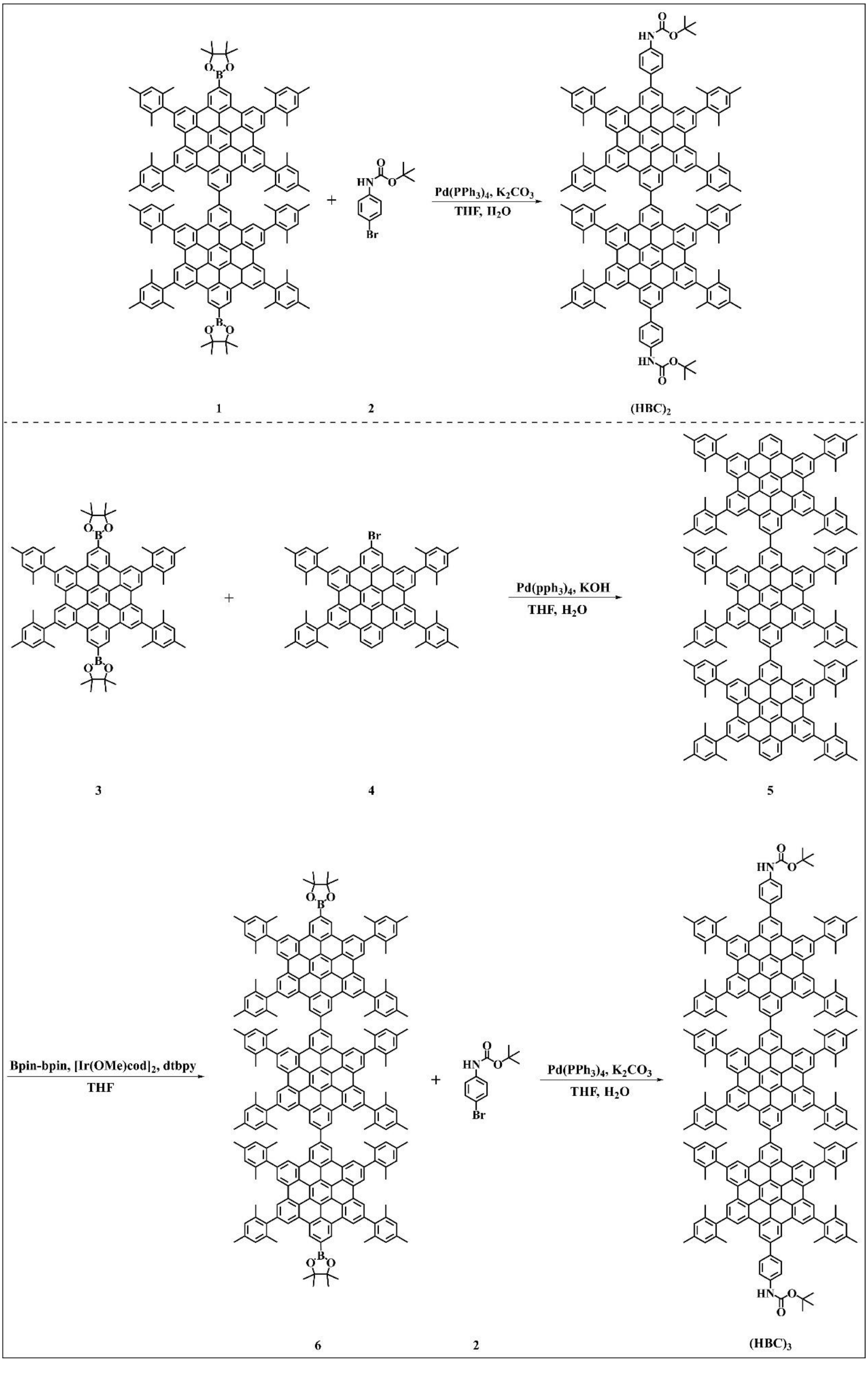

Scheme S1. Synthesis routes of $(HBC)_2$ and $(HBC)_3$. Reprinted with permission from

Ref. 2. Copyright 2024 American Chemical Society.

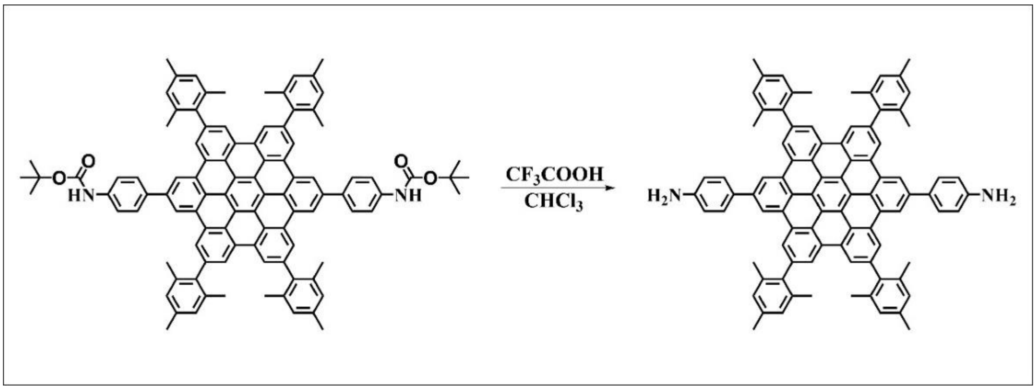

Scheme S2. The reaction route for removing the BOC groups. Reprinted with permission from Ref. 2. Copyright 2024 American Chemical Society.

Synthesis of compound **1**: The synthesis of **1** is based on the published papers[3].

Synthesis of compound **2**: The synthesis of **2** is based on the published papers[4].

Synthesis of compound $(HBC)_2$: A mixture of **1** (540 mg, 0.24 mmol), **2** (400 mg, 1.47 mmol), and K_2CO_3 (350 mg, 2.53 mmol) is placed in a round-bottom flask (100 mL), to which THF (15 mL) and H_2O (2 mL) are added. After purging with Ar for 15 minutes, $Pd(PPh_3)_4$ (20 mg, 7%) is added, and the mixture is purged with Ar for another 15 minutes before sealing the apparatus. The mixture is then heated to 70°C and stirred at this temperature for 48 h in the dark. Most of the solvent is removed, and the residue is extracted with DCM and dried over $MgSO_4$. The product is purified by chromatography on a silica gel column using DCM/petroleum ether (2:1) as the eluent. The yield is 515 mg (90%). ^{1}H NMR (400 MHz, $CDCl_3$) δ (ppm) showed 9.76 (s, 4H), 9.45 (s, 4H), 9.30 (s, 4H), 9.22 (s, 4H), 9.11 (s, 4H), 9.09 (s, 4H), 8.03 (d, J = 8.5 Hz, 4H), 7.64 (d, J = 8.2 Hz, 4H), 7.18 (s, 8H), 7.11 (s, 8H), 6.67 (s, 2H), 2.49 (s, 12H), 2.44 (s, 12H), 2.30 (s, 24H), 2.27 (s, 24H), 1.60 (s, 18H) (Figure S1); HRMS (MALDI-TOF) analysis revealed a *m*/*z* calcd for $C_{178}H_{140}N_2O_4$ $[M]^+$ of 2371.0880; found 2371.1896 (Figure S2). Note that the procedures of molecular synthesis are reprinted with permission from Ref. 2. Copyright 2024 American Chemical Society.

Synthesis of compound **3**: The synthesis of **3** is based on the published papers[5].

Synthesis of compound **4**: The synthesis of **4** is based on the published papers[3].

Synthesis of compound **5**: A mixture of **3** (150 mg, 0.12 mmol), THF (20 mL), H2O (2 mL), **4** (260 mg, 0.24 mmol), and KOH (50 mg, 0.89 mmol) is added to a round-bottom flask (100 mL). The mixture is purged with Ar for 15 minutes. $Pd(PPh_3)_4$ (20 mg, 7%) is then added, and the mixture is purged with Ar for an additional 15 minutes. The mixture is heated to 70°C and stirred in the dark for 48 h. Most of the solvent is removed, and the residue is extracted with DCM and dried over $MgSO_4$. The product is purified by chromatography on a silica gel column using DCM/petroleum ether (1:3) as the eluent. The yield is 320 mg (89%). The ^1H NMR (400 MHz, $CDCl_3$) δ (ppm) showed 9.78 (s, 8H), 9.36-9.23 (m, 12H), 9.18-9.04 (m, 16H), 8.25 (t, J = 8.3 Hz, 2H), 7.16 (s, 8H), 7.12 (s, 16H), 2.48 (s, 12H), 2.44 (s, 24H), 2.33-2.22 (m, 72H) (Figure S3). Note that the procedures of molecular synthesis are reprinted with permission from Ref. 2. Copyright 2024 American Chemical Society.

Synthesis of compound **6**: A mixture of **5** (300 mg, 0.10 mmol), 4,4'-di-tert-butyl-2,2'-bipyridine (2 mg, 7 mol%), $[Ir(OMe)COD]_2$ (4 mg, 6 mol%), and anhydrous THF (10 mL) is added to a sealed tube (25 mL). After purging with Ar for 0.5 hours, bis(pinacolato)diboron (130 mg, 1.0 mmol) is added. The mixture is then purged with Ar for an additional 10 minutes. The mixture is heated to 90°C and stirred in the dark for 48 hours. Most of the solvent is removed, and the residue is thoroughly washed with MeOH (20 mL×3). The product is purified by filtration, resulting in a yellow powder with a yield of 305 mg (94%).The ^1H NMR (400 MHz, $CDCl_3$) δ (ppm) indicated 9.79-9.73 (m, 8H), 9.67 (s, 4H), 9.31-9.24 (m, 12H), 9.13-9.04 (m, 12H), 7.17 (s, 8H), 7.11 (s, 16H), 2.48 (s, 12H), 2.43 (s, 24H), 2.33-2.21 (m, 72H), 1.52 (s, 24H) (Figure S4). Note that the procedures of molecular synthesis are reprinted with permission from Ref. 2. Copyright 2024 American Chemical Society.

Synthesis of compound $(HBC)_3$: The synthesis of $(HBC)_3$ is performed following the same procedure as for $(HBC)_2$, resulting in a yield of 73%. The ^1H NMR (400 MHz,

$CDCl_3$) δ (ppm) showed 9.81-9.75 (m, 8H), 9.45 (s, 4H), 9.31 (d, J = 3.7 Hz, 8H), 9.22 (s, 4H), 9.15-9.07 (m, 12H), 8.03 (d, J = 8.5 Hz, 4H), 7.64 (d, J = 7.3 Hz, 4H), 7.18 (s, 8H), 7.12 (s, 16H), 6.66 (s, 2H), 2.48 (s, 12H), 2.44 (s, 24H), 2.32-2.27 (m, 72H), 1.60 (s, 18H) (Figure S5). HRMS (MALDI-TOF) *m*/*z* calculated for $C_{256}H_{196}N_2O_4$ $[M]^+$: 3363.5262; is found to be 3362.8850 (Figure S6). Note that the procedures of molecular synthesis are reprinted with permission from Ref. 2. Copyright 2024 American Chemical Society.

Removing BOC groups: 5 mg of the compound with amino–terminated $(HBC)_1$ are to be placed into a round-bottom flask. 5 mL of chloroform solvent are to be added to dissolve it. And 0.5 mL of trifluoroacetic acid is added dropwise under an ice bath. The mixed solution is to be stirred at room temperature for 4 hours, after which an appropriate amount of triethylamine is to be added to neutralize the reaction mixture until the pH is neutral. Dichloromethane and water are then to be added to extract the reaction mixture, and the organic layer solution is to be collected. The solvent is to be removed using a rotary evaporator, and the target product amino–terminated $(HBC)_1$ is obtained. Amino–terminated $(HBC)_1$ is to be immediately used in the reaction for assembling molecules with electrodes.

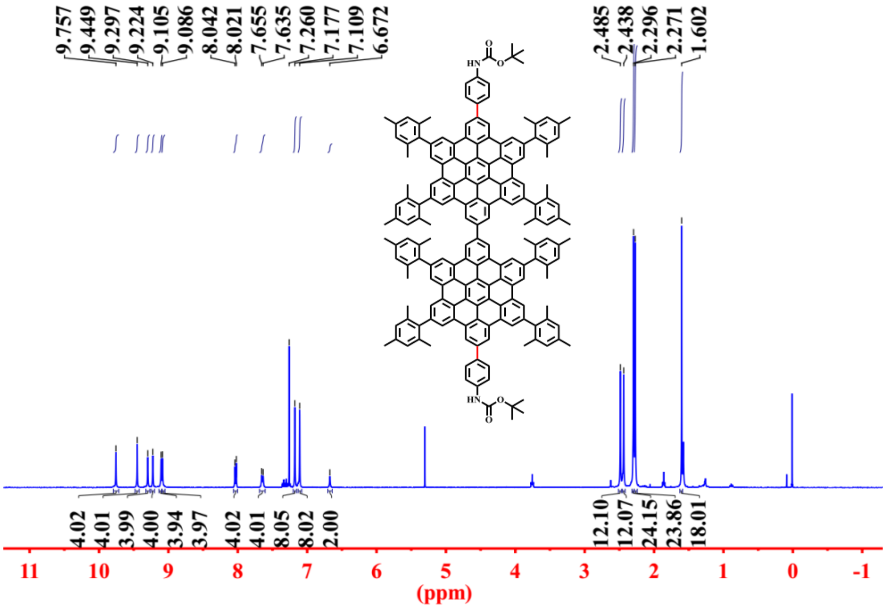

Figure S1. ^{1}H NMR spectrum of $(HBC)_2$ in $CDCl_3$. Reprinted with permission from

Ref. 2. Copyright 2024 American Chemical Society.

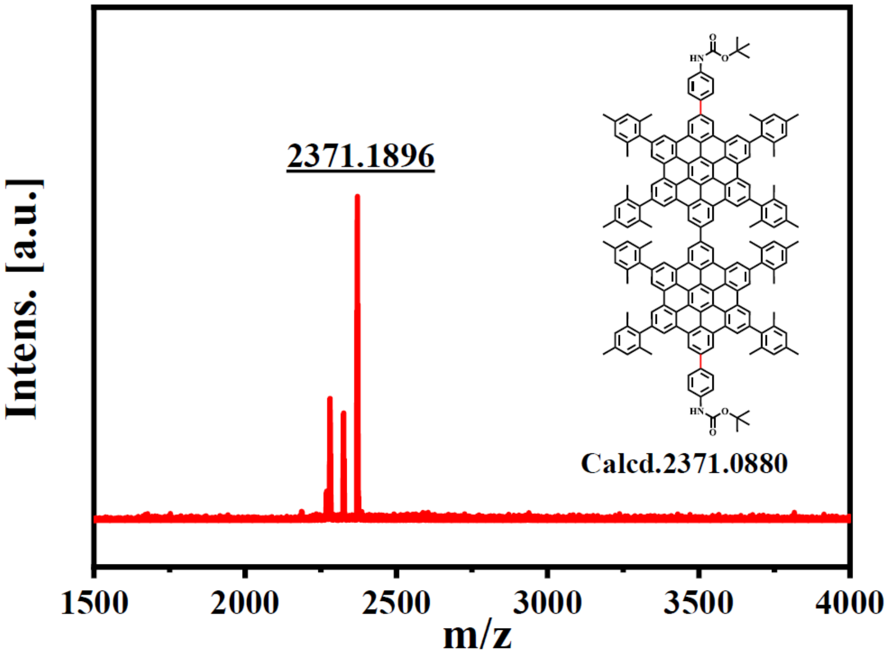

Figure S2. HRMS (MALDI-TOF) data for $(HBC)_2$-BOC. Reprinted with permission from Ref. 2. Copyright 2024 American Chemical Society.

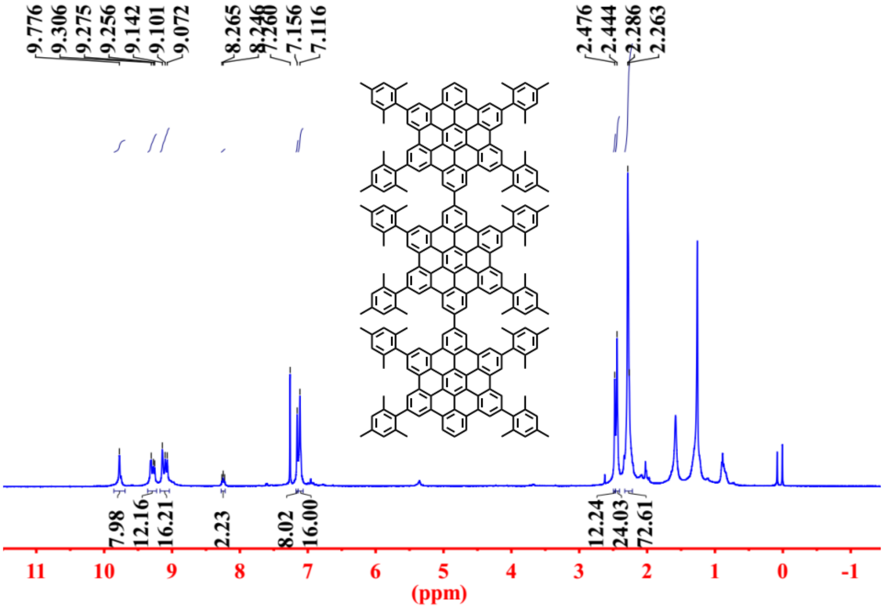

Figure S3. ^{1}H NMR spectrum of **5** in $CDCl_3$. Reprinted with permission from Ref. 2. Copyright 2024 American Chemical Society.

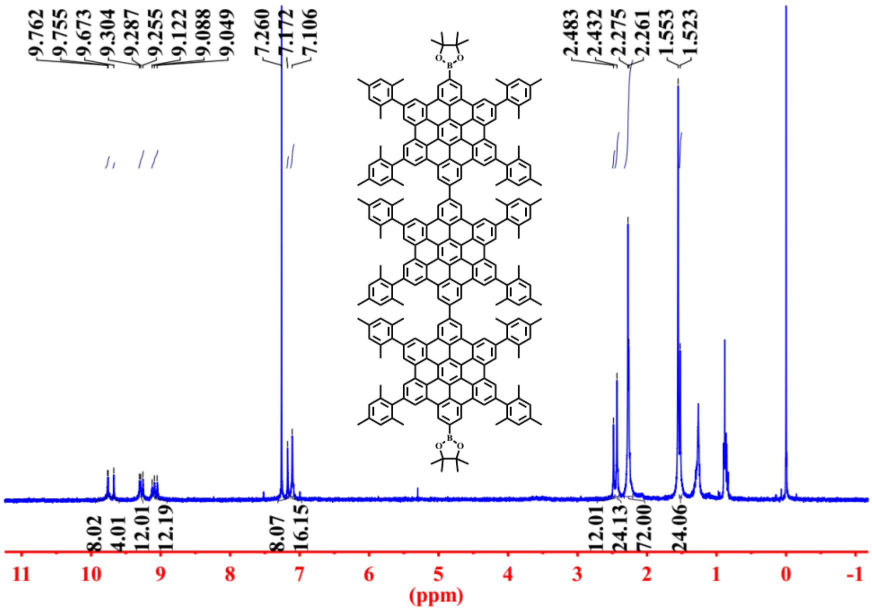

Figure S4. ^{1}H NMR spectrum of **6** in $CDCl_3$. Reprinted with permission from Ref. 2. Copyright 2024 American Chemical Society.

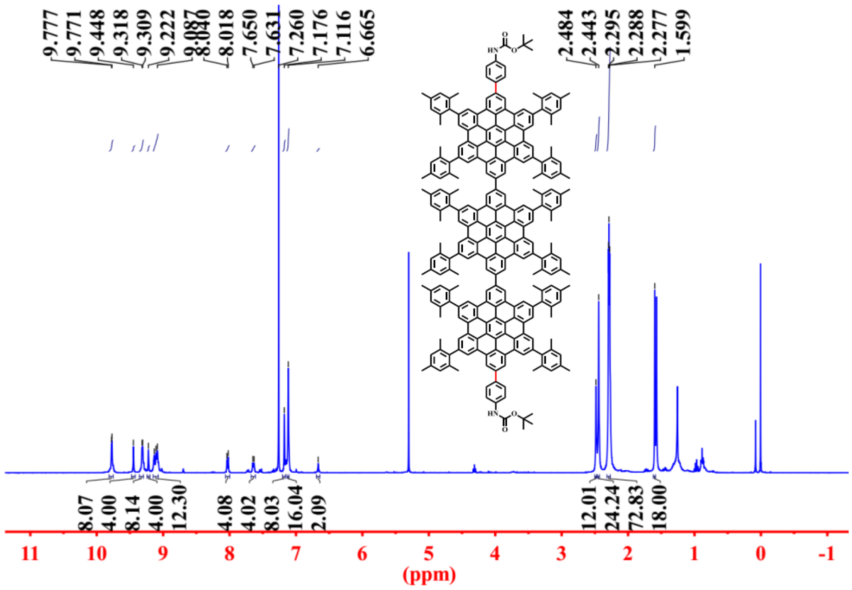

Figure S5. ^{1}H NMR spectrum of $(HBC)_3$-BOC in $CDCl_3$. Reprinted with permission from Ref. 2. Copyright 2024 American Chemical Society.

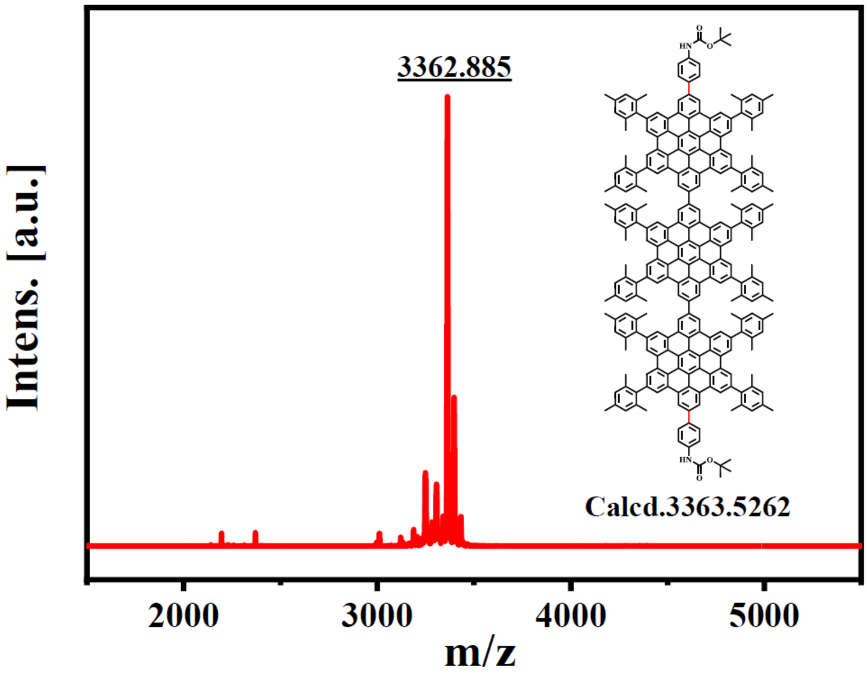

Figure S6. HRMS (MALDI-TOF) data for $(HBC)_3$-BOC. Reprinted with permission from Ref. 2. Copyright 2024 American Chemical Society.

2. Device fabrication

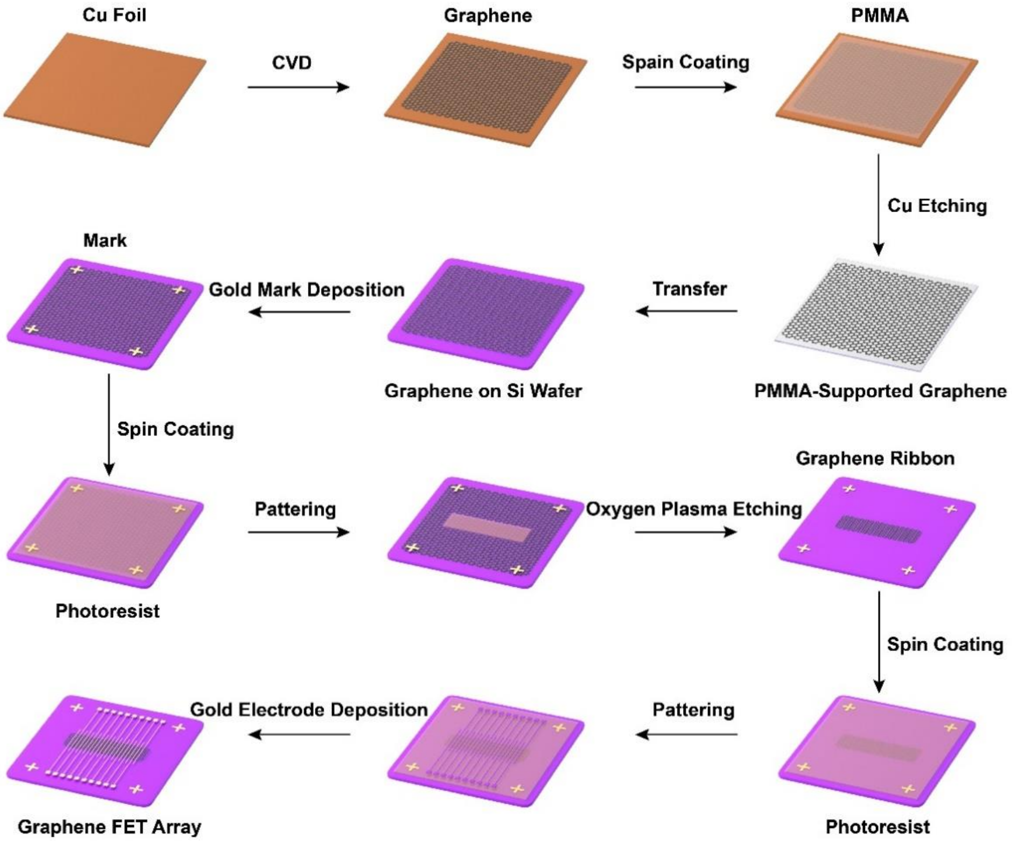

Figure S7. Fabrication process of graphene FET array device.

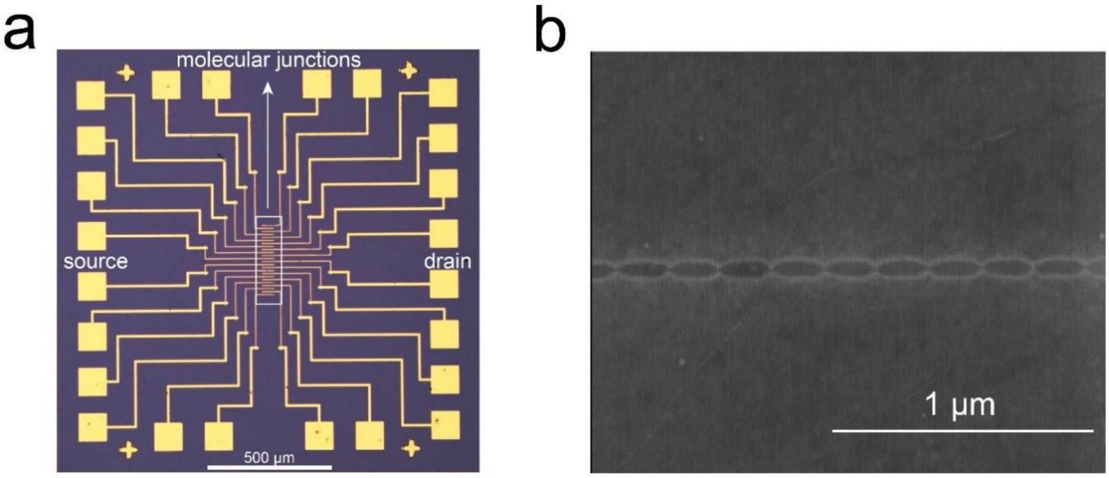

Figure S8. a. Optical image of device of GMG-SMJs. **b**. SEM images of representative indented graphene point contact array.

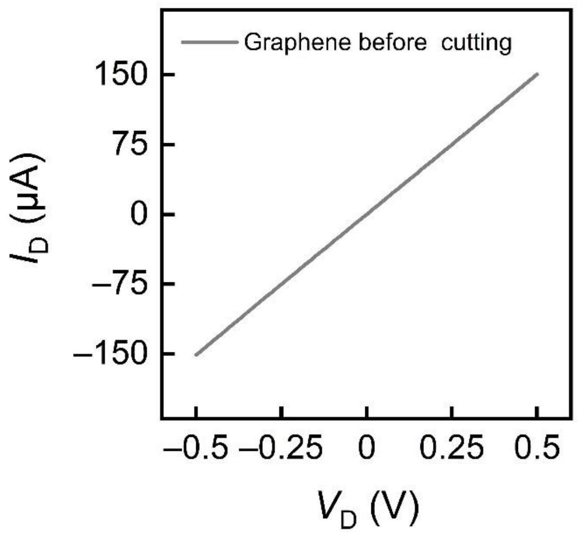

Figure S9. *I–V* characteristic curves of the graphene before cutting. This curve indicates that the device has good conductance.

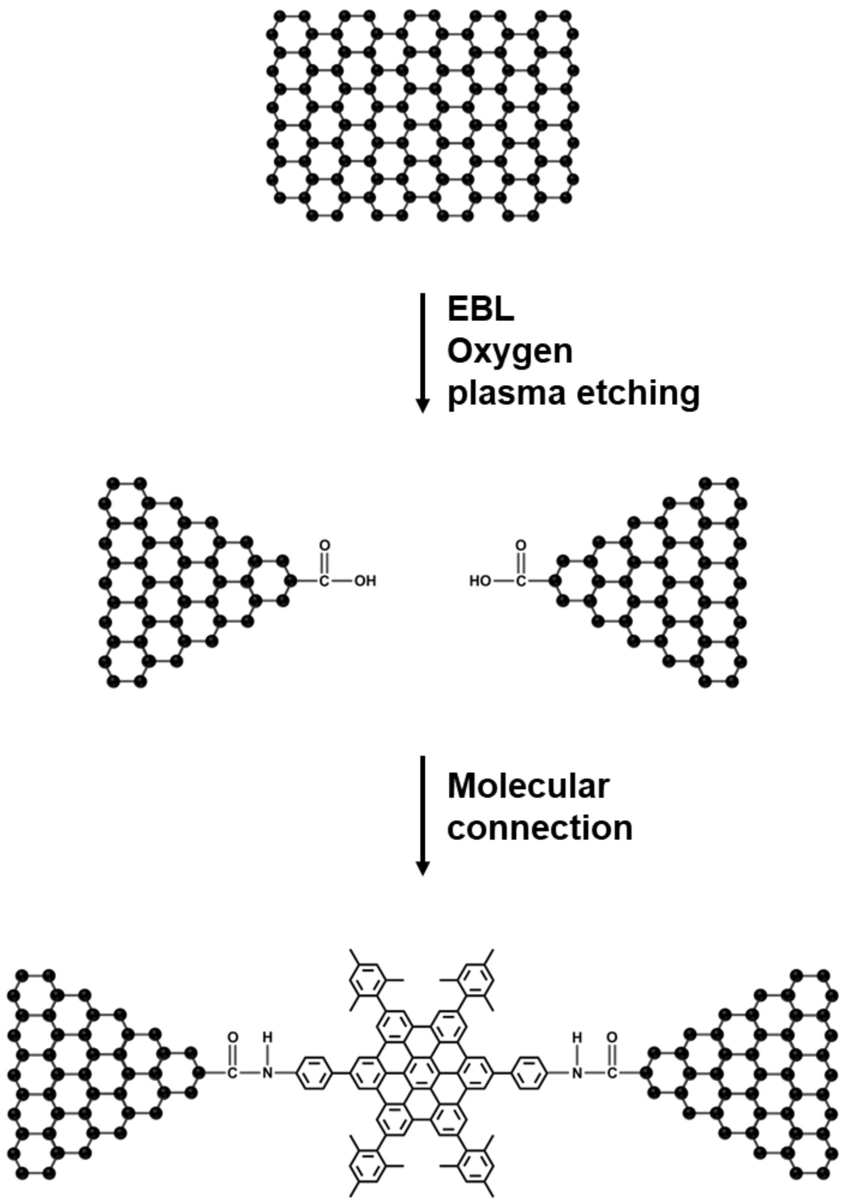

Figure S10. Preparation of graphene electrodes and schematic diagram of connecting single HBC molecules between graphene electrodes.

3. Device measurements

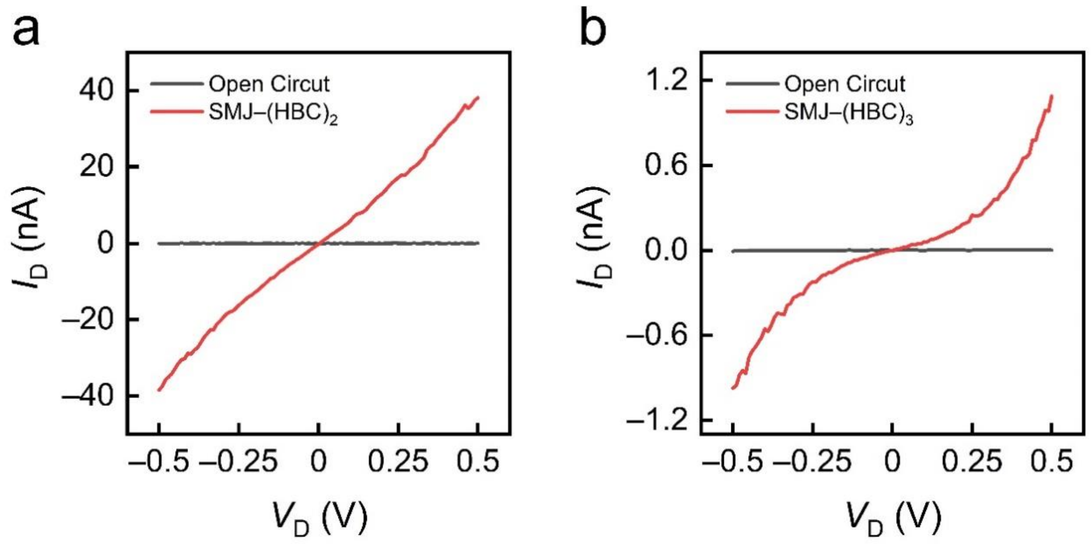

Figure S11. The *I–V* characteristic curves of open circuit graphene nanoelectrodes, and after connecting single molecules of $(HBC)_2$ and $(HBC)_3$.

4. Characterization of single-molecule junctions

Inelastic tunneling spectra (IETS) of single-molecule junctions: The IETS measurements are conducted at a temperature of 2 K with an alternating voltage of 10 mV and a frequency of 331 Hz. The IETS can detect chemical bonding information in the single-molecule junction, where *v*(C=O, ~220 mV) and *v*(N−H, ~450 mV) correspond to the stretching vibrations of C=O and N−H, respectively. This indicates that the molecular terminals are covalently bonded to graphene electrodes through amide bonds.

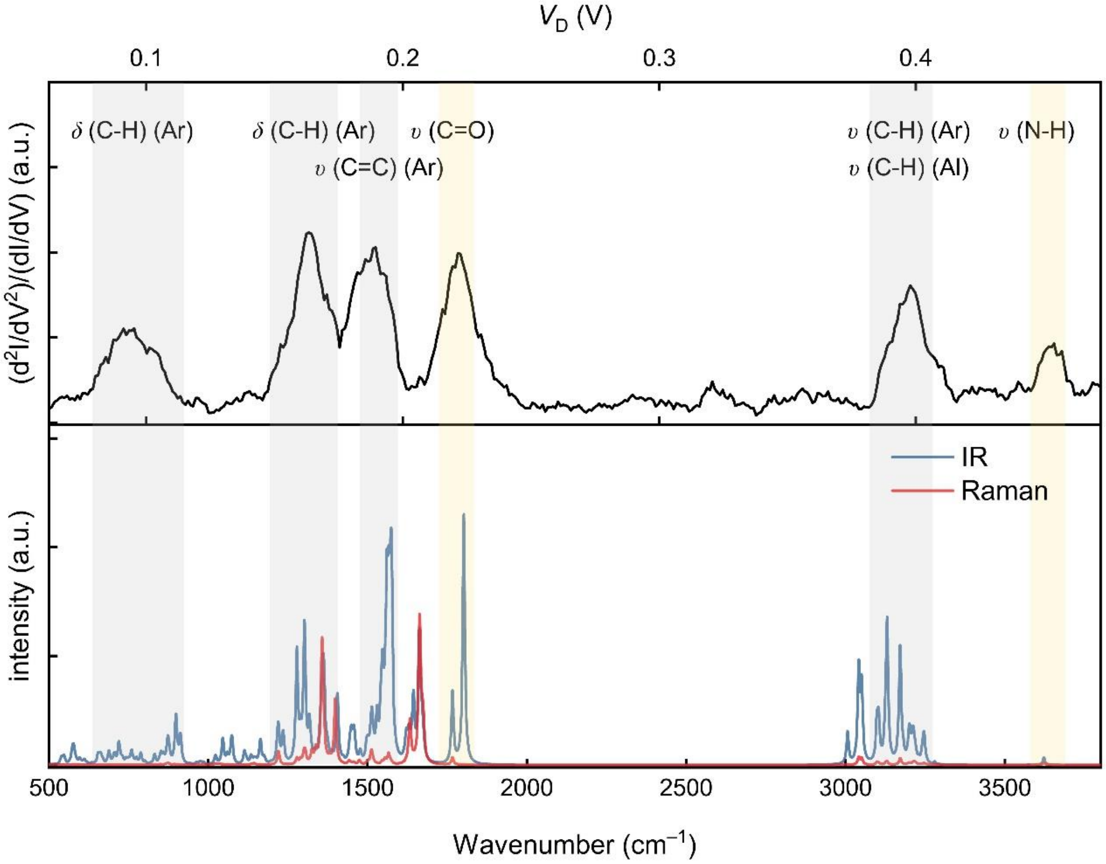

Figure S12. Top: high-resolution IETS for $(HBC)_1$-based single-molecule junctions measured by a lock-in second-harmonic technique at 2.0 K with an alternating voltage modulation of 10 mV (rms value) at a frequency of 311 Hz. Bottom: corresponding calculated infrared and Raman spectra.

Super–resolution fluorescence: The device is placed under the fluorescence microscope, and a ×100 oil lens is used to precisely focus the laser beam (5 mW, 405

nm) on the graphene device, with an exposure time of 50 ms. Utilizing the STORM (stochastic optical reconstruction microscopy), the super-resolution fluorescence imaging of the single-molecule connection is obtained.

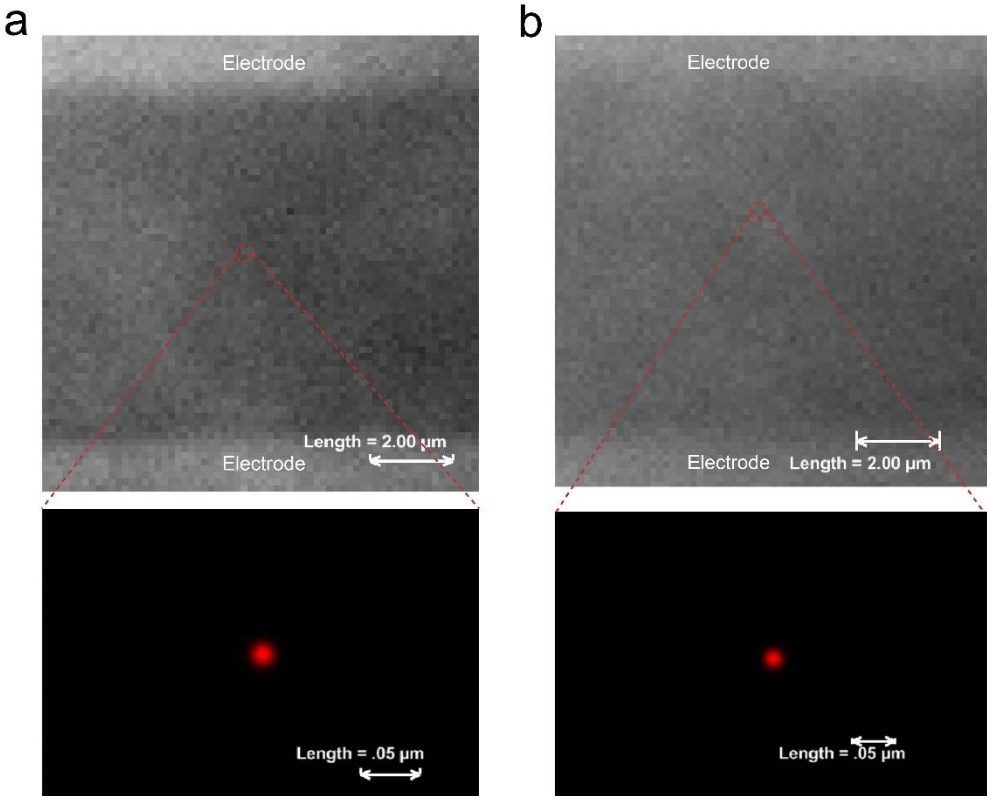

Figure S13. Super–resolution fluorescence imaging of single molecule junctions is obtained by STORM (stochastic optical reconstruction) effect. a. graphene–$(HBC)_2$–graphene. b. graphene–$(HBC)_3$–graphene.

Probabilistic analysis: After undergoing electron beam lithography with dashed lines and oxygen plasma etching, *m* pairs of graphene point electrodes (with 210 intervals, thus *m* equals 210) can be prepared between a pair of gold electrodes. Here, we assume that the probability of a molecule being connected between a pair of graphene point electrodes is *p*. Since the connection of molecules in each nanogap is an independent event, following a binomial distribution, therefore, the probability G_n of having n molecules connected between a pair of gold electrodes is:

$$G_n = \frac{m!}{n!(m-n)!} p^n (1-p)^{m-n} \qquad n = 0,1,2,3,\ldots,m \qquad (1)$$

When a molecule successfully connects any pair of electrodes within the 210 pairs of graphene electrodes, a current signal can be detected, indicating that the molecular junction is conductive. At this point, the success rate of molecular connections can be expressed as:

$$\gamma_{\text{connection}} = 1 - G_0 = 1 - \frac{m!}{0!(m-0!)} p^0 (1-p)^m \qquad (2)$$

where G_0 represents the probability of no molecular connection. By optimizing the experimental conditions, the success rate of molecular connections is approximately $\gamma_{\text{connection}} \approx 10\%$. Using formula (2), we can calculate that $p = 0.000502$. Substituting this value into formula (1), we obtain $G_1 \approx 0.0949$. Consequently, the proportion of the current signal measured between a pair of gold electrodes that originates from a single-molecule junction is $G_1/\gamma_{\text{connection}} \approx 94.9\%$. Based on the above results, we believe that the devices successfully detecting electrical signals have a very high probability of being connected by only a single molecule.

5. Charge transport characteristics

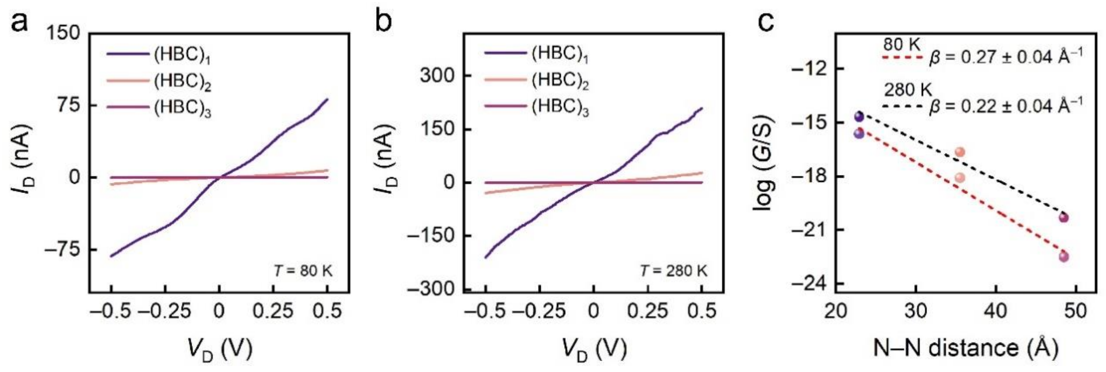

Figure S14. The attenuation of conductance with molecular length for the graphene–(HBC)$_n$–graphene molecular junction.

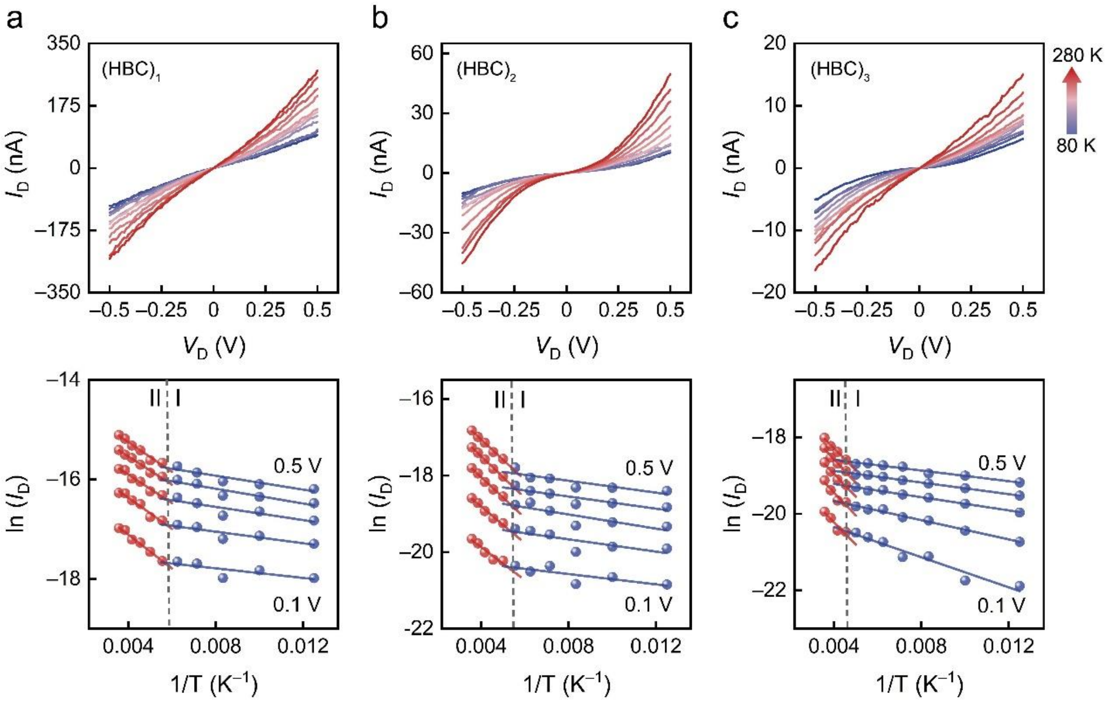

Figure S15. Temperature–dependent charge transport characteristics from another set of devices.

Table S1. The vibrational modes for rotations of benzene and HBC rings in $(HBC)_1$ connected to graphene fragments, modelled as electrodes.

Vibration modes	Frequency (cm^{-1})
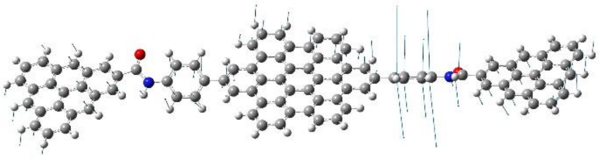	50.88
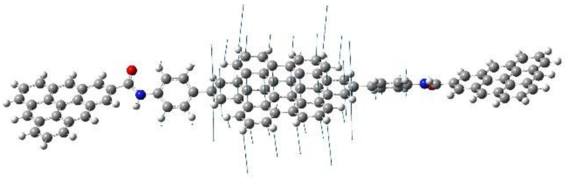	128.46

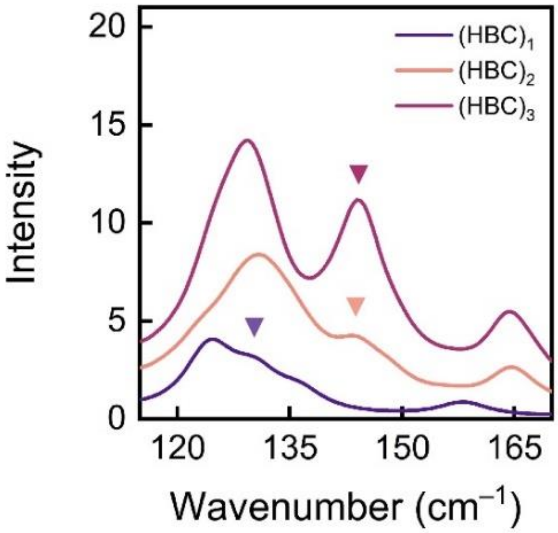

Figure S16. Vibrational spectra of $(HBC)_n$ with all side chains (trimethylbenzene). The vibrational modes of trimethylbenzene couple with the inherent vibrations of the $(HBC)_n$ ring, causing peak splitting. The split peaks are indicated by downward-facing triangles.

Reference

[1] M. Zhang, Z. Wu, H. Jia, P. Li, L. Yang, J. Hao, J. Wang, E. Zhang, L. Meng, Z. Yan, Y. Liu, P. Du, X. Kong, S. Xiao, C. Jia, X. Guo, *Sci. Adv.* **2023**, *9*, eadg4346.

[2] M. Zhang, B. Wang, H. Jia, C. Zhao, J. Hao, W. Liu, L. Zhou, E. Zhang, Y. Chen, P. Du, J. Wang, C. Jia, X. Guo, *ACS Mater. Lett.* **2024**, *6*, 4388.

[3] H. Jia, G. Zhuang, Q. Huang, J. Wang, Y. Wu, S. Cui, S. Yang, P. Du, *Chem. Eur. J.* **2020**, *26*, 2159.

[4] M. Chen, Z.-H. Ren, Y.-Y. Wang, Z.-H. Guan, *J. Org. Chem.* **2015**, *80*, 1258.

[5] R. Yamaguchi, S. Hiroto, H. Shinokubo, *Org. Lett.* **2012**, *14*, 2472.
